# Revised Swedish visual field standards for a driver's licence: Threshold perimetry as a predictor of eligibility according to the current Swedish and current Norwegian suprathreshold standards

**DOI:** 10.1111/aos.70111

**Published:** 2026-03-07

**Authors:** Wid Saadi, Tomas Bro, Susanna Sagerfors

**Affiliations:** ^1^ Department of Ophthalmology Örebro University Hospital Region Örebro County Sweden; ^2^ Department of Biomedical and Clinical Sciences Linköping University Linköping Sweden; ^3^ Department of Ophthalmology Faculty of Medicine and Health, Örebro University Örebro Sweden

**Keywords:** Esterman, threshold perimetry, visual field standards for driving

## Abstract

**Purpose:**

To explore whether threshold perimetry can predict fulfilment of the current Swedish and current Norwegian Esterman perimetry standards for Group 1 driver's licence, and differences in compliance between the former and current Swedish visual field standards.

**Methods:**

This study included perimetry examinations from individuals aged ≥18 years who underwent threshold perimetry (24‐2 or 30‐2) and binocular Esterman visual field testing on the same day during September 2010 to December 2022. Data were collected over 12 years from three Departments of Ophthalmology in Region Örebro County, Sweden. Threshold perimetry data were used to predict fulfilment of the current Swedish and current Norwegian Esterman visual field standards.

**Results:**

In all, 798 individuals were included. Altogether (33%) failed the Swedish visual field standards for a Group 1 driver's licence in the past; by comparison, a significantly smaller proportion would have failed the current Swedish standards today, (18%) (*p* < 0.001; McNemar's test). Threshold perimetry, if all test points within the central 20° had values of ≥10 dB, could predict compliance with a sensitivity of 89% and 88% and a specificity of 73% and 78% for the current Swedish and current Norwegian Esterman standards, respectively.

**Conclusion:**

Threshold perimetry may predict compliance with the current Swedish and current Norwegian Esterman visual field test standard for driving. A larger proportion of individuals may meet the current Swedish visual field standard for a Group 1 driver's licence compared with the former Swedish standard.

## 1 INTRODUCTION

In August 2009, the European Commission issued revised visual standards for a driver's licence (directive 2009/113/EC). According to the directive, Group 1 licence holders must have a binocular visual acuity of at least 0.5, as well as a visual field extending at least 120° laterally and 40° in height (20° up and down) with no defects within a radius of the central 20° (COMMISSION DIRECTIVE 2009/113/EC, 2022). However, the directive does not specify criteria for passing or failing the visual field standards and this likely has contributed to the wide range of implementations by the different member states. A survey on the European visual field standards for driving was performed by a Norwegian research group in 2022 with 25 responding ophthalmic experts from different European countries. Of these, 15 reported that their government endorsed a specific perimetry test to assess visual field standards for driving. The most common approach was a combination of threshold perimetry and Esterman testing (9/15), while three countries relied solely on the Esterman test, two on threshold perimetry test and one on a special two‐level suprathreshold test. Only five of these 15 countries also defined unaccepted losses for compliance of the standards (Sudmann et al., 2022).

The Swedish application of the directive was adopted on 1 September 2010 (TSFS2010:125, 2010). Compared with those of the other Nordic countries, Sweden's regulations, especially the visual field standards, have been considered stricter (Bro & Lindblom, 2018) and perceived by some as unfair, leading to instances of illegal driving and distrust in the licensing authorities (Nyberg et al., 2021).

Recently, Sweden revised its visual field standards, adopting the new regulations on 1 February 2025. The updated standards rely on the Esterman perimetry test (TSFS 2024:65, n.d.), aligning Sweden with Norway, the United Kingdom and Ireland. However, the revised Swedish standards also include an exception: if a threshold perimetry test meets certain criteria, a renewal of a Group 1 driver's licence may be approved without an Esterman perimetry test. In contrast, for licence withdrawal, a failed Esterman perimetry test remains mandatory.

The Esterman test, originally developed by Benjamin Esterman in the 1960s for quantifying visual field function, later evolved into a standardized binocular suprathreshold automated perimetry test (Bro, 2022). In theory, a supra‐threshold test such as an Esterman test, makes no distinction between an individual without the ability to perceive light compared to an individual who can perceive light equivalent to 11 dB, a difference that in several situations, may be significant. Today, the Esterman test is widely used for the evaluation of driving ability and is the approved perimetry test, alone or in combination with threshold perimetry in 12 European countries (Sudmann et al., 2022) despite the lack of correlation between the outcome on Esterman perimetry test and driving performance (Faraji et al., 2022).

However, in clinical practice, static automated threshold visual field perimetry remains the gold standard for the management of, for example, glaucoma (Azuara‐Blanco, 2021).

In both Sweden (Körkortslag 1998:488, n.d.) and Norway (Helsepersonelloven LOV 1999‐07‐02‐64), physicians are required to report patients suspected of being unfit to drive to the relevant authority. The adoption of the current Swedish Esterman visual field standards, together with the existing Norwegian Esterman perimetry standards, may present challenges in routine ophthalmological practice. This is because implementing routine Esterman testing for all patients with visual field defects detected on threshold perimetry could place an unnecessary burden on healthcare resources.

Therefore, it is of clinical value to explore whether threshold perimetry results can indicate when an Esterman perimetry test is warranted to assess compliance with visual field standards for a Group 1 driver's licence.

The objective of this study was to evaluate the impact of the revised Swedish driver's licence visual field standards through three predefined study aims: First, compliance with the revised Esterman‐based standard was compared with compliance under the former combined threshold–Esterman standard. Second, the clinical characteristics of individuals who meet or did not meet the former and revised Swedish visual field standards were characterized. Finally, the study assessed whether static threshold perimetry can identify when an Esterman perimetry test is warranted to assess compliance with the current Swedish and currant Norwegian visual field standards for a Group 1 driver's licence.

## 2 MATERIALS AND METHODS

### 2.1 Study design and ethical approval

This retrospective study was approved by the Swedish Ethical Review Authority, Gothenburg, Sweden (2022‐05822‐01; 2023‐07780‐02).

### 2.2 Study population

The study included individuals ≥18 years of age (18 years being the minimum age to get a Group 1 driver's licence in Sweden and Norway) who underwent both threshold perimetry, irrespectively of thresholding algorithm, SITA standard, fast or faster (24‐2 or 30‐2) for each eye and binocular Esterman perimetry with Humphrey Field Analyser (HFA) (Carl Zeiss AG, Oberkochen, Germany) on the same day between 1 September 2010 and 31 December 2022. Data were collected from three Departments of Ophthalmology in Region Örebro County, Sweden.

Eligible individuals were identified through the Zeiss FORUM ophthalmology software (Carl Zeiss AG, Oberkochen, Germany) and included. If any visual field test for an individual was repeated on the same day, all tests of that individual from that day were excluded to ensure reliability of data, as multiple attempts indicate potential difficulties in test performance. No exclusions of perimetry tests due to the rate of false positive responses or fixation loss were made, since neither the former nor the current Swedish legislation specifies reliability criteria for perimetry testing in the context of licensing.

For each participant, only the earliest (first) perimetry examination within the study period was included. Data collected comprised age at the time of testing, sex, reliability indices and visual field measurement results, including threshold values and the following visual field indices: mean deviation (MD), Visual Field Index (VFI) and Esterman efficiency score (EES).

For each individual, presumed diagnosis on the day of the perimetry test was retrieved from the patient administrative system by searching for ICD‐10 codes related to glaucoma, Nervus opticus or the optic disc, stroke or diabetes. For details on ICD codes used in the diagnosis search, see Supporting Information. For individuals without a recorded diagnosis on the day of examination, an additional search covering the entire study period was performed. The diagnostic code recorded closest to the perimetry test date and considered most likely to indicate the reason for the visual field examination was selected.

### 2.3 Visual field standards

The former Swedish visual field standards (valid from September 2010 to January 2025):

- Threshold perimetry of each eye to assess the visual field within the central 20°.
- Binocular Esterman perimetry test to assess the peripheral visual field, 120 × 40 outside central 20°.

The current Swedish visual field standards (valid from February 2025):

- Binocular Esterman perimetry test to assess both the central and the peripheral visual field.

However, there is an exception in the regulation concerning perimetry test method. Under this exception, a Group 1 driver's licence may be renewed without a binocular Esterman perimetry test. This if all 32 test points within the central 20° of the integrated visual field have a sensitivity of ≥10 dB, and no peripheral visual defects are suspected that would violate the peripheral field standards.

The Norwegian visual field standards (valid from January 2014):

- Binocular Esterman perimetry test to assess both the central and the peripheral visual field

The current Swedish visual field standards for Group 1 driver's licence can be considered less strict than the former, both the central and peripheral standards. Within the central 10°, a sensitivity of ≥10 dB is now accepted compared to ≥20 dB in the former Swedish standards. Both the former and the current Swedish standards accepts one test point outside of the central 10° but within the central 20° to have a value of less than 10 dB. In the periphery one cluster of 3 test points missed on Esterman test, outside the central 20° is accepted, which was not accepted in previous Swedish standards.

The central Norwegian standards can be considered stricter than the current Swedish since in general no loss within the central 20° is accepted. On the other hand, in the periphery, the Norwegian standards accept several clusters consisting of three missed test points, compared to only one cluster of three missed test points according to current Swedish standards.

A detailed description of the former Swedish, current Swedish and current Norwegian visual field standards for a Group 1 driver's licence is provided in the Supporting Information.

### 2.4 Threshold perimetry–based assessment tools

**FIGURE 1:**
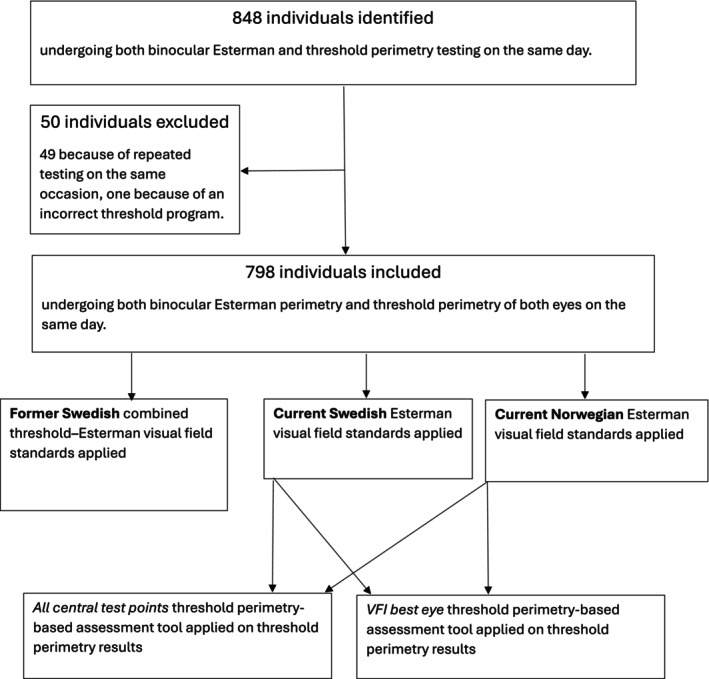
Flowchart of included individuals and perimetry tests.

Two threshold perimetry–based tools (Figure 1) were developed to determine whether threshold perimetry can help identify individuals who may require Esterman testing to rule out non‐compliance, according to current Swedish and Norwegian Esterman perimetry standards for Group 1 drivers

1. *All central test points*: All 32 test points within the central 20° of visual field (integrated visual field) are required to have a sensitivity of ≥10 dB for a ‘pass’ on the assessment tool. ‘Integrated visual field’ refers to the combined test results from both eyes, using the maximum sensitivity for each individual test point (Crabb & Viswanathan, 2005). This assessment tool was chosen because the current Swedish visual field standards contain an exception, which allows the use of integrated visual fields to verify compliance with Esterman criteria if all 32 central test points in the integrated visual field have a sensitivity of at least 10 dB.
2. *VFI better eye*: The cut‐off value for passing was determined using receiver operating characteristic (ROC) curve analysis, optimizing sensitivity and specificity (for the cut‐off, see Section 3).

### Reliability criteria

To study false positive response rate and fixation loss, a cut‐off value of ≥15% for false positive responses and ≥20% for fixation loss was chosen based on when a warning is issued in the Zeiss software (Humphrey Field Analyser instructions for use).

### 2.5 Statistics

#### 2.5.1 Compliance with former and current Swedish and Norwegian visual field standards for group 1 driving Licences

McNemar's test was used to compare the proportion of individuals passing the former vs. the current Swedish visual field standards for a Group 1 driver's licence, in the entire cohort and stratified after diagnosis. Mann–Whitney *U* test was used to compare age, EES, MD (better and worse eye) and VFI (better and worse eye) between individuals who passed or failed the visual field standards of the former and the current Swedish and current Norwegian criteria. Chi‐squared (*χ* ^2^) test or Fisher's exact test (when appropriate) was used to analyse distribution of sex. Statistical calculations were performed in SPSS version 25 (IBM Corp, Armonk, NY, USA).

To enable comparisons between individuals who met the former and the current Swedish standards, a bootstrap‐based test with 1000 resamples was used. This approach was chosen as this analysis included, but was not limited to, paired observations (Thulin, 2024). The analyses were conducted in R version 4.4.3 using the packages boot and boot.pval.

#### 2.5.2 Statistical evaluation of threshold perimetry–based assessment tools

Evaluation of the threshold perimetry–based assessment tools, in terms of sensitivity, specificity, positive predictive value, negative predictive value, Cohen's kappa and positive and negative likelihood ratio, was performed through Vassarstats.net (Lowry, n.d.).

Receiver operating characteristic curve analysis was performed to identify an optimal threshold for *VFI better eye*, maximizing sensitivity and specificity. The *VFI better eye* cut‐off was selected based on ROC analysis, maximizing sensitivity and specificity. Comparison of sensitivity/specificity of the *All central test points* and *VFI better eye* assessment tools in predicting outcomes of the Esterman test for both the whole cohort and subgroups based on diagnosis, was performed according to Kim and Lee (2017), that is, comparing sensitivity and specificity separately for individuals with or without ‘the disease’ in this case passing, respectively, failing the visual field standards. The proportion of false negatives between assessment tools was also compared using McNemar's test. Since the current Swedish regulations allow approval without an Esterman test if all 32 central test points on threshold perimetry (integrated visual field) have a sensitivity ≥10 dB, binary logistic regression analysis was performed to identify variables influencing a false negative outcome on the *All central test points* assessment tool. The variable test duration, right and left eye were categorized into three, according to percentiles, lowest, highest and the two middle ones. These statistical calculations were performed in SPSS version 25 (IBM Corp, Armonk, NY, USA).

## 3 RESULTS

### 3.1 The cohort

During the approximately 12‐year study period, between 2010 and 2022, 848 individuals underwent both binocular Esterman and threshold perimetry of the right and left eye on the same day. Of these, 50 individuals were excluded: 49 because of repetitive testing with the same test method and/or of the same eye on the same day and one because of a threshold perimetry test programme, 24‐2C, not compatible with the former Swedish visual field standards. Altogether 798 individuals were included in the study, all of whom underwent both binocular Esterman and threshold perimetry of the right and left eye during the study (Figure 1).

The study cohort consisted of 75% men. Among the participants, 35% had a glaucoma‐related, 25% a diabetes‐related and 26% a neurological diagnosis. The remaining 14% had a diagnosis related to the optic nerve other than glaucoma or a retinal disorder other than diabetes and various diagnoses of uncertain significance in relation to a risk of acquiring a visual field defect. The median EES was 96, and the median MD better eye and median VFI better eye were −3.4% and 93.5%, respectively (Table 1).

**TABLE 1:** Clinical characteristics of the cohort of individuals (*n* = 798) undergoing both threshold and Esterman perimetry in Region Örebro County, Sweden, in relation to compliance with the Group 1 driver's licence criteria according to former, valid between 1 September 2010 and 31 January 2025, and current Swedish, valid from 01 February 2025, and current Norwegian standards.

		Current Swedish as of 1 February 2025		*p*‐Value	Former Swedish 1 September 2010–31 January 2025		*p*‐Value	Current Norwegian		*p*‐Value	Total (*n* = 798)
				(*U* value)			(*U* value)			(*U* value)	
		Pass (*n* = 655)	Fail (*n* = 143)	[*χ* ^2^; df]	Pass (*n* = 537)	Fail (*n* = 261)	[*χ* ^2^; df]	Pass (*n* = 595)	Fail (*n* = 203)	[*χ* ^2^; df]	
Age (years), median (IQR)		65 (54; 73)	68 (59; 74)	0.003^a^ (*U* = 39 290)	63 (52; 72)	68 (60.5; 75)	<0.001^a^ (*U* = 52 945)	64 (53; 73)	69 (61; 75)	<0.001^a^ (*U* = 47 220)	65 (55; 73)
Sex (male)		498 (76%)	104 (73%)	0.406^b^ [*χ* ^2^ = 0.7; df = 1]	412 (77%)	190 (73%)	0.227^b^ [*χ* ^2^ = 1.5; df = 1]	453 (76%)	149 (73%)	0.434^b^ [*χ* ^2^ = 0.6; df = 1]	602 (75%)
Diagnosi S	Glaucoma (*n* = 276)	215 (78%)	61 (22%)		148 (54%)	128 (46%)		179 (65%)	97 (35%)		276 (35%)
	*N*. opticus/optic disc not glaucoma^c^ (*n* = 19)	15 (79%)	4 (21%)		13 (68%)	6 (32%)		14 (74%)	5 (26%)		19 (2%)
	Diabetes (*n* = 196)	183 (93%)	13 (7%)		166 (85%)	30 (15%)		176 (90%)	20 (10%)		196 (25%)
	Retina or vitreous not diabetes^d^ (*n* = 44)	38 (86%)	6 (14%)		35 (80%)	9 (20%)		37 (84%)	7 (16%)		44 (5%)
	Neurological disorder incl. stroke (*n* = 207)	149 (72%)	58 (28%)		123 (59%)	84 (41%)		136 (66%)	71 (34%)		207 (26%)
	Other^e^ (*n* = 56)	55 (98%)	1 (2%)		52 (93%)	4 (7%)		53 (95%)	3 (5%)		56 (7%)
Esterman efficiency score, median (IQR)		97 (92; 100)	80 (68; 92)	<0.001^a^ (*U* = 13 799)	98 (94; 100)	89 (76; 95)	<0.001^a^ (*U* = 29 348)	98 (93; 100)	85 (72;95)	<0.001^a^ (*U* = 22 350)	96 (88; 99)
Mean deviation better eye, median (IQR)		−2.6 (−5.5; −0.7)	−9.3 (−13.6; −5.9)	<0.001^a^ (*U* = 15 654)	−1.9 (−4.2; −0.4)	−8.4 (−12.3; −5.4)	<0.001^a^ (*U* = 16 293)	−2.3 (−5.1; −0.6)	−8.4 (−12.9; −5)	<0.001^a^ (*U* = 20 531)	−3.4 (−7.1; −1.1)
Mean deviation, worse eye, median (IQR)		−5.8 (−13.1; −2.5)	−16.3 (−21.2; −10.2)	<0.001^a^ (*U* = 20 922)	−4.6 (−9.5; −2.1)	−15.7 (−20.7; −10.3)	<0.001^a^ (*U* = 22 807)	−5.1 (−11.6; −2.3)	−15.7 (−20.7; −10.2)	<0.001^a^ (*U* = 24 093)	−7.6 (−15.5; −3.1)
VFI better eye, %, median (IQR)		96 (89; 99)	75 (62; 83)	<0.001^a^ (*U* = 10 199)	97 (93; 99)	78 (68.5; 86)	<0.001^a^ (*U* = 9113)	96 (91; 99)	78 (66; 86)	<0.001^a^ (*U* = 14 296)	93.5 (83; 98)
VFI worse eye, %, median (IQR)		87 (67; 95)	57 (34; 73)	<0.001^a^ (*U* = 19 145)	91 (78; 96)	57 (36; 72.5)	<0.001^a^ (*U* = 19 973)	90 (71; 96)	57 (37; 73)	<0.001^a^ (*U* = 21 861)	83 (59; 94)

Abbreviations: CI, confidence interval; df, degree of freedom; VFI, Visual Field Index.

^a^ Mann‐Whitney *U* test, *U* in parentheses, rounded to the nearest whole number.

^b^ Chi‐squared (*χ* ^2^) test, rounded to one decimal place and degrees of freedom in square brackets.

^c^ Unspecified changes of the optic nerve head (*n* = 10), optic disc drusen (*n* = 2), optic atrophy (*n* = 1), papillary oedema (*n* = 3), and optic neuritis (*n* = 3).

^d^ Age‐related macular degeneration (*n* = 16), retinal vein/artery occlusion (*n* = 7), hereditary retinal dystrophy (*n* = 7), retinal detachment including retinoschisis (*n* = 6), vitreous haemorrhage/detachment/opacification (*n* = 4), posterior cyclitis/panuveitis (*n* = 2), degenerative myopia (*n* = 1), choroidal melanoma (*n* = 1).

^e^ Diplopia, exophoria, abducens nerve palsy and unspecified paralytic strabismus (*n* = 5), iridocyclitis (*n* = 2), various diagnoses including vertigo, hereditary corneal dystrophy, post‐phaco surgery, cataract, subjective vision loss, lacrimal gland disease (*n* = 20), impaired vision in one eye (*n* = 5), suspicion of restricted visual field or night blindness or subjective loss of visual function (*n* = 17), high myopia, amblyopia and keratoconus (*n* = 4), commanded by authority or general practitioner (*n* = 3).

**FIGURE 2:**
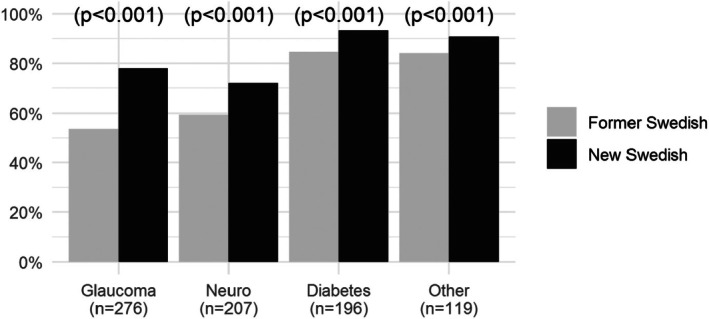
Pass rate according to former Swedish and current Swedish visual field standards for Group 1 driver's licence subgrouped according to diagnosis. For comparison, McNemar's test was used.

A significantly larger proportion would have fulfilled the current Swedish standards, compared to the former Swedish standards both in the entire cohort (82% vs. 67%, *p* < 0.001; McNemar's test and when stratifying for the three most common diagnosis), glaucoma (78% vs. 54%, *p* < 0.001; McNemar's test), diabetes (93% vs. 85%, *p* < 0.001; McNemar's test) and neurological disorders, including stroke (72% vs. 59%, *p* < 0.001; McNemar's test) (Figure 2). Among the 261 individuals who failed the former Swedish standards, 49% would have met the current Swedish standards (Table 1).

The group fulfilling the current standards had a significantly lower VFI worse eye (*p* = 0.012) and Mean Deviation better eye (*p* = 0.003) worse eye (*p* = 0.001) (Table S1).

### 3.2 Threshold perimetry–based tools for assessing compliance with current Swedish and Norwegian Esterman‐based visual field standards

To evaluate the predictive performance of threshold perimetric measures, ROC curves were generated using the number of test points within the central 20° with a value of ≥10 dB (integrated visual field), and VFI. These were assessed for their ability to predict fulfilment of the current Swedish and current Norwegian visual field standards. Among the tested parameters, central test points of at least 10 dB and VFI of the better eye yielded the highest AUC for both the current Swedish (0.889; 95% CI 0.854–0.925 and 0.891; 95% CI 0.864–0.918) and current Norwegian (0.890; 95% CI 0.859–0.920 and 0.882; 95% CI 0.855–0.908) standards.

Based on the ROC curve analysis, optimal cut‐off values were established for passing with the assessment *tool VFI better eye*. For the current Swedish standards, a VFI of the better eye of ≥86% was required to pass the assessment tool, whereas a value of ≥89% is needed for the Norwegian standards (Figure S1).

### 3.3 Threshold‐based assessment tools in relation to revised Swedish Esterman visual field standards

A significant difference was observed between the two threshold perimetry‐based assessment tools *All central test points* and *VFI better eye* when evaluated using McNemar's test with stratification according to Kim and Lee (Kim & Lee, 2017) (Table 2). The *VFI better eye* tool demonstrated significantly higher specificity (83%; 95% CI 80–85) compared with the *All central test points* tool (73%; 95% CI 69–76). However, the proportion of false negatives was significantly lower for *All central test points* than for *VFI better eye* (2% vs. 3.5%; *p* < 0.023; McNemar's test) (Table 2). Both assessment tools exhibited a negative predictive value exceeding 95%. Among the individuals with a glaucoma diagnosis (*n* = 276), *VFI better eye* had a significantly higher specificity (73%; 95% CI 67–79) than did *All central test points* (53%; 95% CI 46–59) (Table S2).

**TABLE 2:** Comparison between two threshold perimetry‐based assessment tools to assess compliance with the current Swedish and the current Norwegian Esterman perimetry standards for a Group 1 driver's licence in a cohort of 798 individuals.

	Current Swedish Esterman perimetry standards for a group 1 driver's licence			Current Norwegian Esterman perimetry standards for a group 1 driver's licence		
	All central test points^a^	VFI better eye^b^	*p*‐Value	All central test points^a^	VFI better eye^b^	*p*‐Value
			(Pass *n* = 655)			(Pass *n* = 595)
			[Fail *n* = 143]			[Fail *n* = 203]
Sensitivity (%) (95% CI (%))	88.8% (82.2%–93.3%)	80.4% (72.8%–86.4%)	(*p* < 0.001) [*p* = 0.023]^c^	87.7% (82.2%–91.7%)	81.8% (75.6%–86.7%)	(*p* < 0.271) [*p* < 0.029]^c^
Specificity (%) (95% CI (%))	72.7% (69.1%–76.0%)	82.7% (79.6%–85.5%)		78.5% (74.9%–81.7%)	80.5% (77.0%–83.6%)	
Positive predictive value (%) (95% CI (%))	41.5% (36.0%–47.3%)	50.4% (43.8%–57.1%)		58.2% (52.4%–63.7%)	58.9% (52.9%–64.6%)	
Negative predictive value (%) (95% CI (%))	96.7% (94.7%–98.1%)	95.1% (92.9%–96.7%)		94.9% (92.5%–96.6%)	92.8% (90.2%–94.8%)	
Positive likelihood ratio (95% CI)	3.25 (2.83–3.73)	4.66 (3.87–5.62)		4.08 (3.47–4.79)	4.19 (3.52–5.00)	
Negative likelihood ratio (95% CI)	0.15 (0.10–0.24)	0.24 (0.17–0.33)		0.16 (0.11–0.23)	0.23 (0.17–0.30)	
Prevalence of failure to meet Group 1 driver's licence criteria (95% CI)	0.18 (0.15–0.21)	0.18 (0.15–0.21)		0.25 (0.22–0.29)	0.25 (0.22–0.29)	
False negative (*n*) (%)	16 (2%)	28 (3.5%)	<0.023^d^	25 (3.1%)	37 (4.6%)	0.029^d^

Abbreviations: CI, confidence interval; VFI, Visual Field Index.

^a^ *All central test points screening tool*: A sensitivity threshold of ≥10 dB at all 32 test points within the central 20‐degree integrated visual field threshold perimetry was selected as the passing criterion for the screening assessment tool.

^b^ *VFI better eye screening tool*: A cut‐off of ≥86% was selected for the assessment tool to predict compliance with the current Swedish Esterman standards, and a cut‐off of ≥89% to assess compliance with the current Norwegian Esterman standards, according to ROC curve analysis.

^c^ McNemar's test stratified the cohort, in accordance with Kim and Lee (2017), into individuals who passed the current Swedish/current Norwegian standards (in parentheses) and individuals who failed (in square brackets).

^d^ McNemar's test.

Of the 16 individuals who passed according to the *All central test points* assessment tool but failed the current Swedish Esterman standards, eight (50%) failed the central standards and eight (50%) the peripheral standards. None failed both central and peripheral standards.

A binominal logistic regression was performed to assess the influence of diagnosis, sex, threshold perimetry test duration and threshold reliability indices (false positive responses and fixation loss) on a false negative outcome of the *All central test points* assessment tool. All independent variables were categorical. Three cases had a studentized residual >3 but were retained in the analysis. Eight cases had leverage values ranging from 0.208 to 0.310 and six had a Cook's distance of >1 (1.03–1.68), all of whom were also retained in the analysis.

The regression model itself was statistically significant (*χ* ^2^(14) = 28.1; *p* < 0.014). The Hosmer and Lemeshow test was not significant (*χ* ^2^(8) = 8.8; *p* = 0.356). The model explained 22% (Nagelkerke *R* ^2^) of the variance in false negative prediction of the *All central test points* screening tool in predicting fulfilment of the current Swedish visual field standards. Only one of the six predictors was statistically significant: a ≥15% false‐positive rate in both eyes, which increased the odds 13.1‐fold of passing the *All Central Test Points* test despite not meeting the current Swedish visual field standard (Table S3).

### 3.4 Threshold‐based assessment tools in relation to current Norwegian Esterman visual field standards

A significant difference between *All Central Test Points* and *VFI Better Eye* was found only among individuals who failed the Norwegian standard (*p* = 0.029), with no difference among those who passed (*p* = 0.271). No other significant differences were observed.

However, the proportion of false negatives, that is, individuals who passed the assessment tool but failed the Esterman perimetry standards, was significantly lower for *All central test points* (3.1%; 25/798) than for *VFI better eye* (4.6%; 37/798) (*p* = 0.029; McNemar's test) (Table 2).

Both tests had a negative predictive value exceeding 92% (Table S4).

Among the 25 individuals who passed on the *All central test points* assessment tool but failed to meet the current Norwegian Esterman standards, two (8%) failed both the central and the peripheral Norwegian standards, 17 (68%) failed the central only and the remaining six (24%) the peripheral standards only.

## 4 DISCUSSION

A higher proportion of individuals in this cohort of 798 met the current Swedish visual field standards, compared to previous standards. Among those previously at risk of licence withdrawal due to visual field defects, nearly half would now meet the revised standards.

Men were overrepresented in the cohort of the present study. This may reflect the sex distribution of older Swedish driver's licence holders. In a previous Swedish study, Ståhl et al. found a pronounced difference in sex distribution among driver's licence holders, with a male to female ratio of 7:2 between the age of 65–69 and a ratio of 7:1 among those between 70 and 74 years of age (Ståhl 1984). In a more recent Spanish study, a similar sex distribution with male overrepresentation, 77% among driver's licence holders, age 65–79 was shown (Mirabet et al., 2023).

In both Sweden and Norway, physicians are legally required to report individuals who do not fulfil the legislative standards for driving. This study explores whether threshold perimetry can predict pass/fail outcomes on Esterman perimetry, based on the current Swedish and current Norwegian Esterman visual field standards for Group 1 driver's licence.

Two assessment tools, ‘*All central test points’* and ‘*VFI better eye’* were developed using threshold perimetry data. Both tools performed well in predicting pass/fail according to current Swedish and current Norwegian Esterman standards, given that the sum of sensitivity and specificity exceeded 1.5 (150%) for both tools (Power et al., 2013), they may be considered useful for clinicians in both Sweden and Norway.

A high false positive rate is less concerning since a ‘fail’ on the assessment tool defined on threshold perimetry will prompt further testing with an Esterman test, due to the current legislation in Sweden and Norway.

The most important aspect for the assessment tools in this study is that a pass on the assessment tool is equivalent to fulfilment of the current Swedish and current Norwegian Esterman standards. Both assessment tools performed well in this regard, with negative predictive values of ≥95% and ≥92%. In Sweden, an exception in the legislation, allows a pass on ‘*All central test points’* assessment tool to replace Esterman perimetry testing when assessing eligibility for continued possession of Group 1 driver's licence. However, in the present study, the ‘*VFI better eye’* tool demonstrated higher specificity than ‘*All central test points’* both in the full cohort and in the subgroup of patients with glaucoma.

Our findings contrast with those of Crabb et al., who studied 65 individuals with primary open‐angle glaucoma. Their assessment tool, based on integrated visual field threshold perimetry for the English visual field standards, had no false negatives; all individuals who passed their assessment test also passed the Esterman standards (Crabb et al., 2004).

Several factors may contribute to this discrepancy. Unlike the British study (Crabb et al., 2004), perimetry test results were not excluded based on reliability indices, since the Swedish legislation, both the former and current does not define when a perimetry test result, threshold nor Esterman is considered reliable for licensing. Additionally, the English cohort may have had more severe visual field defects, and the cut‐off values for threshold‐perimetry assessment tool differed slightly, as did the Swedish and English Esterman standards.

When the English threshold assessment test was applied to individuals with a paracentral scotoma of various aetiologies the sensitivity of threshold perimetry in predicting fulfilment or not of English Esterman visual field standards was 86% (Chisholm et al., 2008). This is comparable to the sensitivities of 89% and 88% for the ‘*All central test’ point* assessment tool in predicting outcome of the current Swedish and current Norwegian Esterman standards in the present study.

Since lack of central defects could only explain 50% (*n* = 8/16) and 24% (*n* = 6/25) of the false negative individuals on *All central test points* for the current Swedish and the Norwegian visual field standards, respectively, we performed a binominal logistic regression analysis. The regression model could explain 22% of the variance of a false negative outcome on the assessment tool with only one statistically significant variable, namely a ≥15% false positive response rate, in both eyes, on threshold perimetry. We therefore conclude that other factors may influence, such as a slightly different head positioning when undergoing threshold test compared to Esterman test in the HFA.

A strength of the current study is the size of the study cohort; another strength is that the included individuals had undergone both perimetry tests, static threshold perimetry of both eyes and binocular Esterman test, on the same day. Limitations are the retrospective study design, with risk of less precision in the information on the diagnosis mainly responsible for the visual field defect. In this study, no exclusion of visual field tests due to reliability indices were made, since neither the former nor the current Swedish legislation includes a definition of a reliable perimetry test in terms of licensing. Not excluding tests with a high false positive response may be considered a limitation of this study and may have overestimated the number of individuals who pass the visual field standards. Including tests irrespective of reliability indices may increase uncertainty in the agreement between threshold perimetry assessment tools and the Swedish and Norwegian Esterman‐based visual field standards. Selecting individuals who have undergone Esterman perimetry may introduce a selection of individuals at risk/suspected of not meeting the former Swedish standards. This means that the proportion of individuals not fulfilling the former Swedish visual field standards may be overrated in the cohort. However, it may also be underrated since failure to meet the former central visual field threshold perimetry standards, made Esterman testing of the peripheral standards superfluous. However, in terms of studying assessment tools, neither sensitivity nor specificity is influenced by the prevalence of the condition, that is, failure to meet the visual field standards for a Group 1 driver's licence (Šimundić, 2009).

In conclusion, the current Swedish Esterman visual field standards for a Group 1 driver's licence may fail fewer individuals than did the former Swedish standards. Analysing the central 32 test points of the integrated visual field in threshold perimetry, assuming a false positive rate of less than 15%, may aid the ophthalmologist in decision making when an Esterman perimetry test is needed to rule out failure to meet the current Swedish and current Norwegian visual field standards. However, since Esterman perimetry has been shown to correlate poorly with driving performance, the challenge in the future is to find strategies to identify individuals who, due to visual field defects, are not suitable drivers.

## FUNDING INFORMATION

The funding organization had no role in the design or conduct of this research.

## CONFLICT OF INTEREST STATEMENT

No conflicting interests exist for any of the authors.

## Supporting information


Figure S1.

Appendix S1.

Table S1.

Table S2.

Table S3.

Table S4.
