# Supplementary material for: Revised Swedish visual field standards for a driver's licence: Threshold perimetry as a predictor of eligibility according to the current Swedish and current Norwegian suprathreshold standards
Source: Acta Ophthalmol. 2026 Mar 7;104(6):e709–17. doi: 10.1111/aos.70111 (PMC13460349; doi:10.1111/aos.70111)
Supplement: Supplementary file 1 [file AOS-104-e709-s002.pdf]

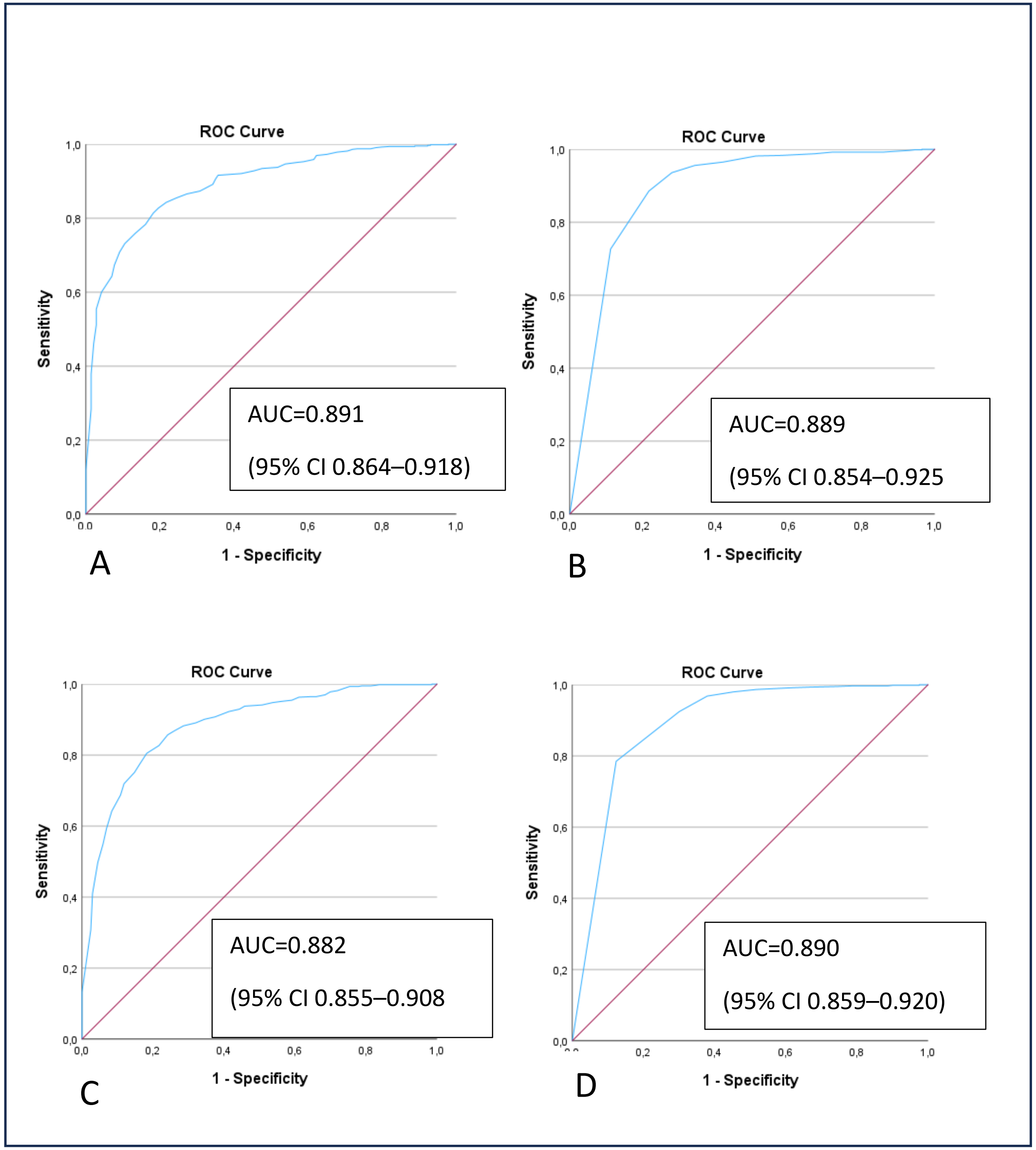
ROC Curve
Sensitivity
1 - Specificity
AUC=0.891
(95% CI 0.864–0.918)
A
ROC Curve
Sensitivity
1 - Specificity
AUC=0.889
(95% CI 0.854–0.925
B
ROC Curve
Sensitivity
1 - Specificity
AUC=0.882
(95% CI 0.855–0.908
C
ROC Curve
Sensitivity
1 - Specificity
AUC=0.890
(95% CI 0.859–0.920)
D

Figure S1 Receiver-operating characteristic (ROC) curves showing compliance with current Swedish and current Norwegian Esterman visual field standards in relation to two variables from static threshold perimetry, Visual Field Index (VFI) of the better eye, and central test point integrated visual field with values of ≥10 dB.

(A) *VFI better eye* threshold perimetry to predict compliance with current Swedish Esterman visual field standards; (B) test points within 20 degrees of ≥10 dB integrated visual field – threshold perimetry to predict compliance with current Swedish Esterman visual field standard; (C) *VFI better eye* threshold perimetry to predict compliance with current Norwegian Esterman visual field standards; (D) test points within 20 degrees of ≥10 dB integrated visual field – threshold perimetry to predict compliance with current Norwegian Esterman visual field standards.

AUC = area under the curve; CI = confidence interval.
