# Supplementary material for: Revised Swedish visual field standards for a driver's licence: Threshold perimetry as a predictor of eligibility according to the current Swedish and current Norwegian suprathreshold standards
Source: Acta Ophthalmol. 2026 Mar 7;104(6):e709–17. doi: 10.1111/aos.70111 (PMC13460349; doi:10.1111/aos.70111)
Supplement: Supplementary file 2 [file AOS-104-e709-s001.docx]

| Table S1 Comparison between individuals fulfilling former Swedish vs current Swedish visual field standards for Group 1 driver’s licence respectively by a nonparametric bootstrap, with 1000 resamples. | | | |
| --- | --- | --- | --- |
|  | *Fulfilling former Swedish standards(n=537)* | *Fulfilling current Swedish standards (n=655)* | *p-value* |
| Age, (yrs) median | 63.0 | 65.0 | 0.432 |
| Esterman efficiency score, median | 98 | 97 | 0.564 |
| Mean deviation, better eye, median | -1.9 | -2.6 | 0.003 |
| Mean deviation worse eye, median | -4.6 | -5.8 | 0.001 |
| VFI better eye, %, median | 97 | 96 | 0.052 |
| VFI worse eye, %, median | 91 | 87 | 0.012 |

VFI = Visual Field Index
