# Supplementary material for: Revised Swedish visual field standards for a driver's licence: Threshold perimetry as a predictor of eligibility according to the current Swedish and current Norwegian suprathreshold standards
Source: Acta Ophthalmol. 2026 Mar 7;104(6):e709–17. doi: 10.1111/aos.70111 (PMC13460349; doi:10.1111/aos.70111)
Supplement: Supplementary file 3 [file AOS-104-e709-s003.docx]

| Table S2 Comparison between two threshold perimetry-based assessment tools for compliance with the current Swedish Esterman visual field standards, for Group 1 driver’s licence, in relation to diagnosis. | | | | | | | | | |
| --- | --- | --- | --- | --- | --- | --- | --- | --- | --- |
|  | Glaucoma (n=276) | | | Diabetes (n=196) | | | Neurological disorder (n=207) | | |
|  | *All central test points*^1^ | *VFI better eye*^2^ | p-value  (Pass n=215)  [Fail n=61] | *All central test points*^1^ | *VFI better eye*^2^ | p-value  (Pass n=183)  [Fail n=13] | *All central test points*^1^ | *VFI better eye*^2^ | p-value  (Pass n=149)  [Fail n=58] |
| Sensitivity (95% CI) | 93.4%  (83.3–97.9%) | 86.9%  (75.2–93.8%) | (p<0.001)  [p=0.289]^3^ | 61.5%  (32.3–84.9%) | 84.6%  (53.7–97.3%) | (p=0.383)  [p=0.375]^3^ | 91.4%  (80.3–96.8%) | 74.1%  (60.7–84.4%) | (p=0.035)  [P=0.002]^3^ |
| Specificity (95% CI) | 52.6%  (45.7–59.4%) | 73.5%  (67.0–79.1%) |  | 82.5%  (76.1–87.6%) | 85.2%  (79.1–89.9%) |  | 79.9%  (72.3–85.8%) | 85.9%  (79.0–90.9%) |  |
| Positive predictive value (95% CI) | 35.8%  (28.5–43.9%) | 48.2%  (38.6–57.9%) |  | 20%  (9.6–36.1%) | 28.9%  (16.0–46.1%) |  | 63.9%  (52.5–73.9%) | 67.2%  (54.2–78.1%) |  |
| Negative predictive value (95% CI) | 96.6%  (91.0–98.9%) | 95.2%  (90.4–97.7%) |  | 96.8%  (92.3–98.8%) | 98.7%  (95.0–99.8%) |  | 96.0%  (90.4–98.5%) | 89.5%  (83.0–93.8%) |  |
| Positive likelihood ratio (95% CI) | 1.97  (1.69–2.30) | 3.28  (2.57–4.18) |  | 3.52  (2.07–5.99) | 5.73  (3.77–8.71) |  | 4.54  (3.26–6.31) | 5.26  (3.44–8.04) |  |
| Negative likelihood ratio (95% CI) | 0.12  (0.05–0.32) | 0.18  (0.09–0.34) |  | 0.47  (0.23–0.93) | 0.18  (0.05–0.65) |  | 0.11  (0.05–0.25) | 0.30  (0.19–0.47) |  |
| Prevalence of failure to meet Group 1 driver’s licence criteria (95% CI) | 0.22  (0.17–0.28) | 0.22  (0.17–0.28) |  | 0.07  (0.04–0.11) | 0.07  (0.04–0.11) |  | 0.28  (0.22–0.35) | 0.28  (0.22–0.35) |  |
| False negative (n) | 4 | 8 | 0.289^4^ | 5 | 2 | 0.375^4^ | 5 | 15 | 0.002^4^ |

^1^ *All central test points assessment tool*: A sensitivity threshold of ≥10 dB at all 32 test points within the central 20-degree integrated visual field threshold perimetry was selected as the passing criterion for the screening assessment tool.

*^2^VFI better eye assessment tool*: A cut-off of ≥86% was selected for the assessment tool to predict compliance with the current Swedish Esterman standards, and a cut-off of ≥89% to assess compliance with the current Norwegian Esterman standards, according to ROC curve analysis.

^3^McNemar’s test stratified the cohort, in accordance with Kim and Lee (2017), into those who passed the current Swedish/current Norwegian standards (in parentheses) and those who failed (in square brackets).

^4^McNemar’s test.

CI = confidence interval; VFI = Visual Field Index.

Kim S, & Lee W (2017): Does McNemar's test compare the sensitivities and specificities of two diagnostic tests? *Stat Methods Med Res* 26: 142–154. <https://doi.org/10.1177/0962280214541852>
