# Supplementary material for: Revised Swedish visual field standards for a driver's licence: Threshold perimetry as a predictor of eligibility according to the current Swedish and current Norwegian suprathreshold standards
Source: Acta Ophthalmol. 2026 Mar 7;104(6):e709–17. doi: 10.1111/aos.70111 (PMC13460349; doi:10.1111/aos.70111)
Supplement: Supplementary file 4 [file AOS-104-e709-s004.docx]

| Table S4 Comparison between two threshold perimetry-based assessment tools for a Group 1 driver’s licence compliance according to the current Norwegian Esterman visual field standards, in relation to glaucoma, diabetes or neurological disorder. | | | | | | | | | |
| --- | --- | --- | --- | --- | --- | --- | --- | --- | --- |
|  |  | | |  | | |  | | |
|  | Glaucoma (n=276) | | | Diabetes (n=196) | | | Neurological disorder (n=207) | | |
|  | *All central test points*^1^ | *VFI better eye*^2^ | p-value  (Pass n=179)  [Fail n=97] | *All central test points*^1^ | *VFI better eye*^2^ | p-value  (Pass n=176)  [Fail n=20] | *All central test points*^1^ | *VFI better eye*^2^ | p-value  (Pass n=136)  [Fail n=71] |
| Sensitivity (95% CI) | 92.8%  (85.2–96.8%) | 83.5%  (74.3–90.0%) | (p<0.092)  [p<0.022]^3^ | 65%  (40.9–83.6%) | 85%  (61.1–96.0%) | (p=0.523)  [p=0.219]^3^ | 90.1%  (80.2–95.6%) | 81.7%  (70.4–89.5%) | (p=0.804)  [p<0.031]^3^ |
| Specificity (95% CI) | 61.5%  (53.9–68.5%) | 68.7%  (61.3–75.3%) |  | 84.7%  (78.3–89.5%) | 82.4%  (75.8–87.5%) |  | 86.0%  (78.8–91.2%) | 87.5%  (80.5–92.3%) |  |
| Positive predictive value (95% CI) | 56.6%  (48.5–64.4%) | 59.1%  (50.4–67.3%) |  | 32.5%  (19.1–49.2%) | 35.4%  (22.6–50.6%) |  | 77.1%  (66.3–85.3%) | 77.3%  (65.9–85.9%) |  |
| Negative predictive value (95% CI) | 94.0%  (87.6–97.3%) | 88.5%  (81.7–93.1%) |  | 95.5%  (90.6–98.0%) | 98.0%  (93.7–99.5%) |  | 94.4%  (88.3–97.5%) | 90.2%  (83.4–94.4%) |  |
| Positive likelihood ratio (95% CI) | 2.41  1.98–2.92 | 2.67  (2.11–3.37) |  | 4.23  (2.64–6.80) | 4.83  (3.34–6.98) |  | 6.45  (4.22–9.86) | 6.54  (4.13–10.33) |  |
| Negative likelihood ratio (95% CI) | 0.12  (0.06–0.24) | 0.24  (0.15–0.38) |  | 0.41  (0.23–0.75) | 0.18  (0.06–0.52) |  | 0.11  (0.06–0.23) | 0.21  (0.13–0.34) |  |
| Prevalence of failure to meet Group 1 driver’s licence criteria (95% CI) | 0.35  (0.30–0.41) | 0.35  (0.30–0.41) |  | 0.10  (0.06–0.16) | 0.10  (0.06–0.16) |  | 0.34  (0.28–0.41) | 0.34  (0.28–0.41) |  |
| False negative (n) | 7 | 16 | 0.022^4^ | 7 | 3 | 0.219^4^ | 7 | 13 | 0.031^4^ |

^1^ *All central test points assessment tool*: A sensitivity threshold of ≥10 dB at all 32 test points within the central 20-degree integrated visual field threshold perimetry was selected as the passing criterion for the screening assessment tool.

*^2^VFI better eye assessment tool*: A cut-off of ≥86% was selected for the assessment tool to predict compliance with the current Swedish Esterman standards, and a cut-off of ≥89% to assess compliance with the current Norwegian Esterman standards, according to ROC curve analysis.

^3^McNemar’s test stratified the cohort into those who passed the current Swedish/current Norwegian standards (in parentheses), and those who failed (in square brackets), in accordance with Kim and Lee (2017).

^4^McNemar’s test.

CI = confidence interval; VFI = Visual Field Index.

Kim S, & Lee W (2017): Does McNemar's test compare the sensitivities and specificities of two diagnostic tests? *Stat Methods Med Res* 26: 142–154. <https://doi.org/10.1177/0962280214541852>
