# Supplementary material for: Revised Swedish visual field standards for a driver's licence: Threshold perimetry as a predictor of eligibility according to the current Swedish and current Norwegian suprathreshold standards
Source: Acta Ophthalmol. 2026 Mar 7;104(6):e709–17. doi: 10.1111/aos.70111 (PMC13460349; doi:10.1111/aos.70111)
Supplement: Supplementary file 5 [file AOS-104-e709-s005.docx]

| **Table S3 Binary logistic regression for the likelihood of predicting a false negative outcome with the *ALL central test points* threshold perimetry-based assessment tool for compliance with the current Swedish Esterman perimetry standards, Group 1 driver’s licence** | | | | | | | | |
| --- | --- | --- | --- | --- | --- | --- | --- | --- |
|  | B | SE | Wald | df | Sig. | Exp(B) | 95% CI for Exp(B) | |
|  |  |  |  |  |  |  | Lower | Upper |
| False positive response rate <15%^1^ in both eyes |  |  | 8.310 | 2 | 0.016 |  |  |  |
| False positive response rate ≥15%^1^ in one eye | 0.136 | 0.869 | 0.025 | 1 | 0.875 | 1.146 | 0.209 | 6.290 |
| False positive response rate ≥15%^1^ in both eyes | 2.573 | 0.910 | 7.994 | 1 | 0.005 | 13.106 | 2.202 | 78.006 |
| Fixation loss <20%^1^ in both eyes |  |  | 2.391 | 2 | 0.303 |  |  |  |
| Fixation loss ≥20%^1^ in one eye | 0.938 | 0.757 | 1.534 | 1 | 0.216 | 2.555 | 0.579 | 11.275 |
| Fixation loss ≥20%^1^ in both eyes | 1.184 | 0.800 | 2.191 | 1 | 0.139 | 3.266 | 0.681 | 15.660 |
| Gender (male) | 1.064 | 0.868 | 1.503 | 1 | 0.220 | 2.899 | 0.529 | 15.893 |
| No known diagnosis with relevance for visual field defect^2^ |  |  | 2.978 | 5 | 0.703 |  |  |  |
| Retinal and vitreous disorders except diabetes^3^ | 18.492 | 4848.292 | 0.000 | 1 | 0.997 | 107425689.577 | 0.000 | – |
| Diabetes | 17.823 | 4848.292 | 0.000 | 1 | 0.997 | 55025172.093 | 0.000 | – |
| Nervus opticus disorder except glaucoma^4^ | 0.317 | 10074.538 | 0.000 | 1 | 1.000 | 1.373 | 0.000 | – |
| Neurological disorder | 18.021 | 4848.292 | 0.000 | 1 | 0.997 | 67048392.879 | 0.000 | – |
| Glaucoma | 16.916 | 4848.292 | 0.000 | 1 | 0.997 | 22211233.005 | 0.000 | – |
| Test duration, right eye, ≤25^th^ percentile^5^ (≤208 sec) |  |  | 3.494 | 2 | 0.174 |  |  |  |
| Test duration, right eye, >25^th^ <75^th^ percentile^5^ (208.25 -306 sec) | -0.057 | 0.839 | 0.005 | 1 | 0.946 | 0.945 | 0.183 | 4.893 |
| Test duration, right eye, ≥75^th^ percentile^5^ (>306 sec) | -1.712 | 1.159 | 2.182 | 1 | 0.140 | 0.181 | 0.019 | 1.750 |
| Test duration, left eye, ≤25^th^ percentile^5^ (≤204 sec) |  |  | 0.811 | 2 | 0.667 |  |  |  |
| Test duration, left eye, >25^th^ <75^th^ percentile^5^ (204.25-300.75 sec) | 17.127 | 3167.117 | 0.000 | 1 | 0.996 | 27439103.808 | 0.000 | – |
| Test duration, left eye, ≥75^th^ percentile^5^ (≥301 sec) | 17.723 | 3167.117 | 0.000 | 1 | 0.996 | 49784379.541 | 0.000 | – |
| Constant | -40.037 | 5,791.075 | 0.000 | 1 | 0.994 | 0.000 |  |  |

The model included 714 individuals; 84 from the study cohort were excluded because of missing values on fixation loss.

^1^Threshold perimetry.

^2^Diplopia, exophoria, abducens nerve palsy and unspecified paralytic strabismus (n=4), iridocyclitis (n=2), various diagnoses including vertigo, hereditary corneal dystrophy, post-phaco surgery, cataract, subjective vison loss, lacrimal gland disease (n=18), impaired vision in one eye (n=4), suspicion of restricted visual field or night blindness or subjective loss of visual function (n=16), high myopia, amblyopia and keratoconus (n=4), and commanded by authority or general practitioner (n=3).

^3^Age-related macular degeneration (n=15), retinal vein/artery occlusion (n=5), hereditary retinal dystrophy (n=6), retinal detachment including retinoschisis (n=6), vitreous haemorrhage/detachment/opacification (n=4), posterior cyclitis/panuveitis (n=2), choroidal melanoma (n=1).

^4^Unspecified changes of the optic nerve head (n=8), optic disc drusen (n=2), optic atrophy (n=1), papillary oedema (n=3), optic neuritis (n=3).

^5^The variable test duration is categorized into three, according to percentiles

CI = confidence interval; df = degree of freedom; Exp(B) = exponential value of B; SE = standard error; sig. = significance.
