# Supplementary material for: Revised Swedish visual field standards for a driver's licence: Threshold perimetry as a predictor of eligibility according to the current Swedish and current Norwegian suprathreshold standards
Source: Acta Ophthalmol. 2026 Mar 7;104(6):e709–17. doi: 10.1111/aos.70111 (PMC13460349; doi:10.1111/aos.70111)
Supplement: Supplementary file 6 [file AOS-104-e709-s006.docx]

Supporting information

Visual field criteria for a Group 1 driver’s licence

**Former Swedish combined threshold and Esterman visual field standard, valid from 1 September 2010 to 31 January 2025:**

*The central 20 degrees to be tested with threshold perimetry*

A value of ≥20 dB of the test points (integrated visual field) within the central 10 degrees; and of ≥10 dB outside of the central 10 degrees but within the central 20-degree integrated visual field is required; however, one test point <10 dB between the central 10–20 degrees is accepted.

*Locations outside of the central 20 degrees to be tested with Esterman perimetry*

A horizontal extent of ≥120 degrees, which includes ≥50 degrees in each direction, right and left, and a vertical extent of 20 degrees above and below the median line, is required. Failure corresponding to three or more adjacent test points is not accepted.

**Current Swedish Esterman visual field standards, 1 February 2025**

*The central 20 degrees to be tested with Esterman perimetry*

No defects are accepted within a radius of the central 10 degrees.

At 10–20 degrees from the centre, a failure corresponding to one test point is accepted.

*Locations outside of the central 20 degrees to be tested with Esterman perimetry*

A horizontal extent of ≥120 degrees, which includes ≥50 degrees in each direction, right and left, and a vertical extent of 20 degrees above and below the median line, is required. A maximum of one area, with failure corresponding to a maximum of three adjacent test points, is accepted. Areas corresponding to a maximum of two adjacent test points are accepted.

**Current Norwegian Esterman visual field standards**

*The central 20 degrees to be tested with Esterman perimetry*

Any missed test point within the central 20 degrees is generally considered as not acceptable.

*Locations outside of the central 20 degrees to be tested with Esterman perimetry*

A cluster of four or more missed test points outside of the central 20 degrees is generally regarded as unacceptable.

**ICD-10 codes used when searching for diagnoses from the patients' medical records**

For a glaucoma-related diagnosis: H40.0, H40.0A, H40.0B, H40.0C, H40.0D, H40.0E, H40.0W, H40.0X, H40.1, H40.1A, H40.1B, H40.1C, H40.1D, H40.1W, H40.1X, H40.2, H40.3, H40.4, H40.5, H40.6, H40.8, H40.9, H42.0, H42.8.

For a diagnosis related to N. opticus or the optic disc, excluding glaucoma: H46.9, H47.0, H47.1, H47.2, H47.3, H47.4, H47.5, H47.6, H47.7, H48.0C, H48.0W, H48.1, H48.8, H48.0B, H48.0A, H48.0.

For a diagnosis related to stroke: H46.0, H46.1, H46.2, H46.3, H46.4, H46.5, H46.6, H46.7, H46.8, I60, I61, I62, I63, I64, I65, I66, I67, I68, I69.

For a diabetes-related diagnosis: diabetes type 1 (E10) or type 2 (E11), + H36.0, H36.0A, H36.0B, H36.0X, H36.8.
